# Bidirectional Relationship of Sleep with Emotional and Behavioral Difficulties: A Five-year Follow-up of Finnish Adolescents

**DOI:** 10.1007/s10964-020-01203-3

**Published:** 2020-02-21

**Authors:** Laura Kortesoja, Mari-Pauliina Vainikainen, Risto Hotulainen, Arja Rimpelä, Henrik Dobewall, Pirjo Lindfors, Sakari Karvonen, Ilona Merikanto

**Affiliations:** 1 Centre for Educational Assessment, University of Helsinki, Helsinki, Finland; 2 Faculty of Education and Culture, Tampere University, Tampere, Finland; 3 Faculty of Social Sciences, Unit of Health Sciences, Tampere University, Tampere, Finland; 4 Department of Adolescent Psychiatry, Pitkäniemi Hospital, Tampere University Hospital, Nokia, Tampere, Finland; 5 Social Policy Research Unit, Finnish Institute for Health and Welfare, Helsinki, Finland; 6 SleepWell Research Program, Faculty of Medicine, University of Helsinki, Helsinki, Finland; 7 Mental Health Unit, Finnish Institute for Health and Welfare, Helsinki, Finland; 8 Orton Orthopaedics Hospital, Helsinki, Finland

**Keywords:** Adolescence, Longitudinal study, Emotional and behavioral difficulties, Sleep duration, Sleep problems

## Abstract

The long-term effects of sleep on adolescent psychosocial well-being are mostly unknown, although insufficient sleep has been associated with emotional and behavioral difficulties in cross-sectional studies. With a five-year follow-up of Finnish adolescents (Time 1: *n* = 8834; Mean age = 13 years, 51.1% female, Time 2: *n* = 5315, Mean age = 15 years, 51.6% female, Time 3: *n* = 3712; Mean age = 17 years; 50.2% female), the purpose of this longitudinal study was to investigate the relations between self-reported sleep duration, sleep problems, and emotional and behavioral difficulties during adolescence. Emotional and behavioral difficulties were assessed using The Strengths and Difficulties Questionnaire (SDQ) measuring emotional symptoms, conduct problems, hyperactivity, peer problems and total difficulties. Sleep duration was calculated by counting the hours between self-reported bedtime and wake-up time. Sleep problems were assessed with a single question about the general sleep problems. According to the cross-lagged models for sleep and emotional and behavioral difficulties, the findings of this study indicate a developmental process during adolescence where, firstly, short sleep duration is a stronger predictor for current and prospective emotional and behavioral difficulties than vice versa. Secondly, increased emotional and behavioral difficulties expose adolescents to current and later sleep problems more strongly than reverse. Thus, the results show that short sleep duration predisposed to emotional and behavioral difficulties across adolescence, which then led to more prospective sleep problems. These findings suggest a developmental process where sleep and emotional and behavioral difficulties are intertwined in shaping adolescents’ health.

## Introduction

Adolescence is a developmental period in life during which many biological, psychological, and social factors interact (Nelson et al. 2005). Changes in sleep (Carskadon 2011) and in mental health (Keyes 2006) during this period are reflected in adolescent’s well-being as increased propensity to depressive symptoms, problems in psychosocial functioning, and conduct problems. These aforementioned problems are classified as emotional and behavioral difficulties in childhood and adolescence (World Health Organization 2015), which can be divided to internalizing problems (e.g. depression and anxiety) and externalizing problems (e.g. hyperactivity and peer/conduct problems) (Goodman et al. 2010). Both internalizing problems and externalizing problems are relatively frequent among adolescents, with prevalence estimates varying from 10% to 30% (Merikangas et al. 2010; Patel et al. 2007). Early emotional and behavioral difficulties may lower the quality of adolescent life and even have long-term effects, e.g. externalizing behavior problems may increase the risk for school dropout, family problems, and economic difficulties (Colman et al. 2009).

The association between sleep and mental health in adolescence is well-established. For example, short sleep duration during adolescence is associated with depressed mood and behavioral problems (Wolfson and Carskadon 1998) and physical and emotional health problems (Owens et al. 2014). In addition, late adolescence is characterized by a circadian shift to eveningness (Roenneberg et al. 2007), which associates with increased risk for externalizing problem behavior (Merikanto et al. 2017). However, the complex associations between sleep and psychosocial development from childhood to late adolescence are still largely unknown. Understanding these associations is important as many mental disorders that are prevalent in adulthood (e.g. depression) begin to develop already during adolescence (Costello et al. 2011; Patel et al. 2007). Thus, longitudinal research is required to understand the complex interplay between sleep and mental health and their long-term effects on well-being. The present longitudinal study aims to answer these questions on how adolescent emotional and behavioral difficulties interact with sleep duration and sleep problems across adolescence.

### Sleep Functioning in Adolescence

Both cross-sectional (Olds, Maher, Blunden, & Matricciani 2010) and longitudinal studies (Gradisar et al. 2011) have shown that sleep duration decreases during adolescence. Individual changes in sleep during childhood/adolescence may be hormonal and puberty-related (Carskadon et al. 1998) or environmentally induced, for example increased autonomy on bedtimes, heavier homework loads, working, sports, social and other activities, and use of electronic devices in adolescence (Harbard et al. 2016). Increasing autonomy and early school start time predispose adolescents to shortened sleep duration and sleep problems, such as sleep fragmentation, especially on school days (Lehto et al. 2016). As a result, this can increase daytime tiredness, depressive symptoms, and burnout, leading to poor daytime functioning (Pesonen et al. 2019). Teenage years are also characterized by a circadian shift to eveningness (Roenneberg et al. 2007), with a biological tendency towards later bedtime and the need to compensate this with a later wake-up time. Recently, it has been shown that chronotype, which refers to the genetic tendency towards morningness or eveningness, modifies the objective sleep timing already from childhood to late adolescence (Merikanto et al. 2018). It has also been postulated that sleep length has shortened over the last 100 years due to the cultural and environmental transitions (Keyes et al. 2015).

### Previous Studies on Association between Sleep and Emotional and Behavioral Difficulties during Adolescence

The association of short sleep with problems in psychosocial functioning during adolescence is well established in cross-sectional studies concerning internalizing and externalizing behavior (Becker et al. 2015). Short sleep (Owens et al. 2014) and sleep problems (Tu et al. 2015) are both associated with internalizing and externalizing difficulties in adolescence. In a previous study, short sleep duration on school nights was associated with feelings of sadness and worthlessness, low motivation, anxiety, and thoughts of self-harm in youth (Yeo et al. 2019). Further, circadian preference for eveningness can lead to impaired daytime functioning (Wolfson and Carskadon 1998) and increased internalizing problems (Quach et al. 2018). Previous research has shown that the relation between eveningness and increased internalizing difficulties may be explained by shorter sleep duration and weakened sleep quality (Merikanto et al. 2017). In addition, later bedtimes during the school week are longitudinally associated with shorter sleep duration, worse educational outcomes and emotional distress (Asarnow et al. 2014).

Previous studies suggest that later bedtimes and short sleep duration could play a role in the etiology of depression (Gangwisch et al. 2010) and lower self-esteem (Fredriksen et al. 2004). Short sleep duration may also increase the risk of adolescent anxiety disorders (Roberts and Duong 2017). Sleep problems were a significant predictor of later internalizing difficulties in a follow-up study of children from the age of 4 years until the age of 13 years, but the association was not significant in reverse (Quach et al. 2018). Compared to internalizing difficulties there are fewer studies about the role of sleep in adolescents’ externalizing difficulties (Becker et al. 2015). Previous studies around the topic suggest that short sleep duration is associated with behavioral problems (Liu and Zhou 2002) and difficulties in interpersonal relations, such as peer problems (Roberts et al. 2009). Furthermore, unhealthy sleep practices and sleep problems (e.g. insomnia) are also related to conduct problems and attention-deficit/hyperactivity disorder (Becker et al. 2015). Sleep problems are also associated with loneliness, especially in early and middle adolescents. Adolescent sleep problems may lead to dysfunction of working memory, which can further increase risky behavior in late adolescence (Thomas et al. 2015).

The associations between sleep and psychosocial well-being are rather bidirectional (Pieters et al. 2015); psychosocial factors can influence sleep and sleep may influence psychosocial functioning (Becker et al. 2015). Psychosocial dysfunction can lead to insufficient sleep. For instance, emotional and behavioral difficulties, especially hyperactivity, are associated with later delayed sleep phase in adolescence (Hysing et al. 2018) It is still largely unknown whether sleep is differentially associated with different dimensions of psychosocial difficulties and vice versa (Quach et al. 2018). Thus far, only few studies have investigated the bidirectional relationships between sleep and emotional and behavioral difficulties in a longitudinal design throughout adolescence, even though the need for longitudinal research has been established (Gregory and Sadeh 2012). What is more, most studies have examined shorter time lags between sleep and emotional and behavioral difficulties. However, there are previous studies that examine reciprocal relations between sleep and psychosocial functioning with multiple time points in adolescence. For instance, daily demands that cause stress are associated with receiving less sleep at night (Fuligni and Hardway 2006). Furthermore, short sleep duration was not associated with psychological well-being 6 or 12 months later, while there was evidence for the reverse relationship (Brand et al. 2014). Externalizing problem behavior is associated with later sleep problems during the elementary school transition period and vice versa (Quach et al. 2018). As noted above, adolescence is a critical developmental period with an increase of both sleep and mental health difficulties (Becker et al. 2015). Insufficient sleep puts youth at risk for a range of mental health difficulties. A longitudinal approach in later adolescence and early adulthood is required in order to elucidate the complex interplay between sleep and emotional and behavioral difficulties and their long-term effects on health prospects across critical transition periods.

### Gender Differences in Adolescent Emotional and Behavioral Difficulties and Sleep

Previous research has shown differences in girls and boys concerning the quality and appearance of mental health problems (Wang et al. 2015). A recent research review revealed that internalizing problems may be on the rise in adolescent girls (Bor et al. 2014). Adolescent girls also report more emotional problems (Fink et al. 2015) and less peer and conduct problems than boys (Van Roy et al. 2006). In contrast, Mishina et al. (2018) found boys to have less peer problems compared to girls. Additionally, previous studies have demonstrated that gender might play a role in the development of sleep length; girls sleep less (Patte et al. 2018) and report more sleep problems than boys (Kechter and Leventhal 2018) during adolescence. This can be due to the developmental differences, since females sleep more than males in late adolescence and early adulthood (Maslowsky and Ozer 2014). However, more research is needed to understand the different developmental paths in girls and boys concerning the associations between sleep and psychosocial well-being.

## Current Study

Using cross-lagged models, this study seeks to examine for the first time how adolescents’ sleep duration and sleep problems predict later emotional and behavioral difficulties and vice versa from early to late adolescence within a single study. Thus far, no previous study has explored the longitudinal bidirectional links between emotional and behavioral difficulties and sleep with a follow-up from early to late adolescence. In addition, the present study aims at investigating the continuity of sleep duration, sleep problems and emotional and behavioral difficulties across adolescent years. Considering sleep functioning in adolescence, it is expected that sleep duration decreases throughout adolescence. Based on prior research, sleep problems and emotional and behavioral difficulties are hypothesized to increase from early to late adolescence leading to more emotional and behavioral problems in those with shorter sleep duration and more sleep problems as compared to adolescents with at least 8 h sleep duration per night and less sleep problems. Moreover, this study examines gender differences in sleep and emotional and behavioral difficulties. Based on previous findings concerning to gender differences, girls were expected to have shorter sleep duration, more sleep problems and emotional symptoms but less hyperactivity and conduct problems as compared to boys.

## Methods

### Participants and Procedure

**Fig. 1:**
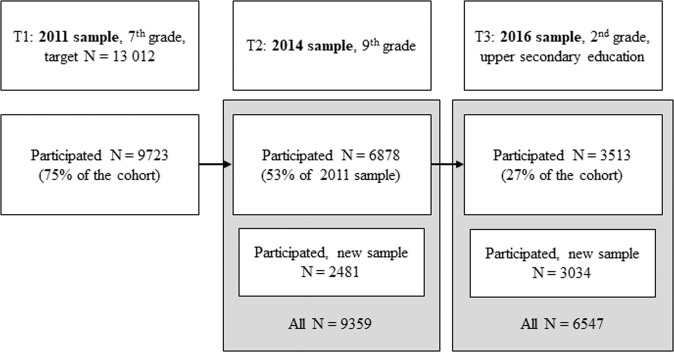
Study flow in the three study years

This study is conducted by Metropolitan Longitudinal Finland (MetLoFin) Study Group focusing on the development of adolescent learning and health. The total number of the age cohort was around 13,500 in 14 municipalities of the Helsinki metropolitan area, observed three times during the years 2011–2016. School data were collected by online classroom surveys, supervised by teachers. All adolescents in the target grade in the participating lower secondary schools of the Helsinki metropolitan area were invited to participate. The three time points were not spaced evenly apart since the study design was not planned primarily for studying sleep but the survey points were based on the transitions points in the Finnish school system. 7th grade was the beginning of the lower secondary school, 9th grade the last grade of the lower secondary school and the last survey point was the second year of the upper secondary school. The original sample was collected in 2011 (T1), when the adolescents were in the seventh grade (12–13 years of age), and a total of 9723 adolescents (50.5% girls) out of the age cohort of around 13,500 students participated in the study. Two follow-ups were done: in 2014 at the end of the ninth grade (T2, 15–16 years of age) and in 2016 in the second year of upper secondary education (T3, 17–18 years of age). At T2, altogether 9359 adolescents (49.2% girls) and at T3, a total of 6547 adolescents (52.4% girls) participated in the MetLoFin study. The study flow is illustrated in Fig. 1. The analytic sample used here consists at T1 of 8743 participants (51.1% girls), at T2 of 5271 (51.6% girls) and at T3 of 3703 (50.2% girls) with information on sleep duration, sleep problems and psychosocial well-being based on The Strengths and Difficulties Questionnaire. The median ages for the analytic samples used here were 13.0 (SD = 0.31) years at T1, 15.0 (SD = 0.49) years at T2, and 17.0 (SD = 0.37) years at T3. A total of 2479 participants (54.0% girls) provided data at every time point (T1, T2, and T3), i.e. around 18.4% of the age cohort.

Adolescents who participated in the study at both T1 and T2 (*n* = 5271) or at both T1 and T3 (*n* = 3703) were compared with adolescents who participated in the study only at T1 (*n* = 2241). Participants who took part in the study only at T1 were more likely to have emotional and behavioral difficulties, to have shorter sleep duration, to be boys, and to have mothers with secondary education or lower (*p* < 0.001 for all independent samples t-tests).

The Ethics Committee of the Finnish Institute for Health and Welfare approved the study design for each data collection. Also, the agency for education in each municipality approved the study every study year. Participation was voluntary for the adolescents. As the research was conducted as part of the school routine, information was collected only on students on the target grade who were present during the school day. Approximately 10% of students are known to be absent each day, some did not want to participate or parents had denied participation. All schools did not want to participate in the surveys or did not distribute the questionnaire to all classes. Parents were also given the right to refuse participation on behalf of their child. In two of 14 municipalities, local authorities required parental consent when the adolescents were 12–13 years of age.

### Measures

#### Sleep duration

Sleep duration was assessed using two questions on sleep and wake-up times. Question 1 asked participants “What time do you usually go to sleep during the school week?” The response scale consisted of seven options: 9 pm or earlier, around 9:30 pm, around 10 pm, around 10:30 pm, around 11 pm, around 11:30 pm, 12 pm or later. Question 2 asked participants “What time you usually wake up during the school week?” The response scale comprised seven options: 7 am or earlier, around 7:30 am, around 8 am, around 8:30 am, around 9 am, around 9:30 am, 10 am or later. Sleep duration was calculated by counting the hours between bedtime and wake-up time. The anchors of bedtime and wake-up time were used to determine estimated sleep duration.

#### Sleep problems

Sleep problems were assessed with a single question: “During the past six months have you had (and if so, how often) difficulty falling asleep or waking up at night?”. The scale contained four response options: seldom or never, approximately once a month, approximately once a week, almost every day.

#### Emotional and behavioral difficulties

The Strengths and Difficulties Questionnaire (SDQ) was used for assessing emotional and behavioral difficulties (Goodman et al. 2010). The SDQ is a psychometric measurement tool for a child’s emotional and behavioral difficulties and has been validated for Finnish school-aged children and adolescents (Koskelainen and Kaljonen 2000). The questionnaire is widely used in the School Health Promotion Study nationwide in Finland. The SDQ includes five subscales that each comprise of five items (responses ranging from 0 to 2): emotional symptoms, conduct problems, hyperactivity, peer problems, and prosocial behavior. The SDQ can be used for measuring both internalizing and externalizing behaviors (Goodman et al. 2010).

#### Total difficulties on psychosocial behavior

The total difficulties on psychosocial behavior includes the first four subscales of The Strengths and Difficulties Questionnaire, excluding the prosocial subscale measuring positive and helpful behavior. Total difficulties score was calculated by summing the scores of the four subscales (range 0–40). The higher the sum score, more total difficulties there were. Cronbach’s alpha coefficients varied over time (*α=* 0.80–0.82) for total difficulties.

#### Emotional symptoms

Emotional symptoms were assessed with items asking if participants had, for example, psychosomatic symptoms (i.e., headaches, belly pain), if they were worried, felt themselves unhappy or were scared of new situations. Emotional symptoms subscale was formed by summing the scores of these items (range 0–10). Cronbach’s alpha coefficients varied over time (α = 0.72–0.75) for emotional symptoms.

#### Conduct problems

Conduct problems subscale measured tendency to lie, stealing things, agreeableness and loosing temper. Conduct problems scores were formed by summing item scores (range 0–10). Cronbach’s alpha coefficients varied over time (*α=* 0.56*–*0.63) for conduct problems.

#### Hyperactivity

Hyperactivity subscale measured restlessness, concentrating, considering before taking action and completing tasks. Hyperactivity scores were formed by summing item scores (range 0–10). Cronbach’s alpha coefficients varied over time (*α=* 0.73–0.75) for hyperactivity.

#### Peer problems

Peer problems subscale measured tendency to do things alone, being bullied, preferring adult companion and popularity of the peer group. Peer problems scores were formed by summing item scores (range 0–10). Cronbach’s alpha coefficients varied over time (*α=* 0.54*–*0.56) for peer problems. For all subscales, higher score indicated more difficulties.

#### Socioeconomic status (SES)

Socioeconomic status (SES) was measured by mother’s educational level. The mother’s educational level was recorded as either secondary education or lower versus tertiary education.

#### Gender

Participants reported their gender as either male or female.

### Data Analyses

**Fig. 2:**
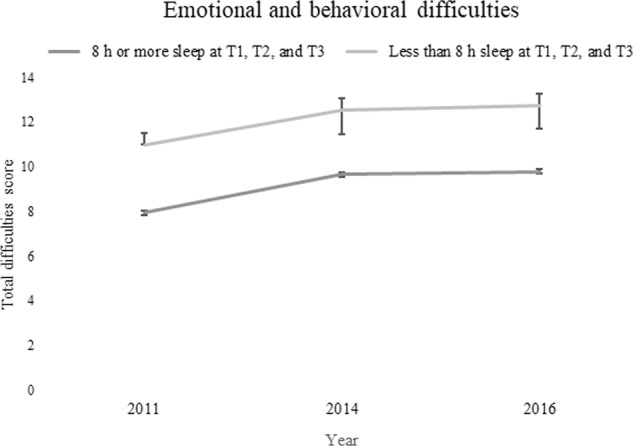
Cross-lagged relationships between emotional and behavioral difficulties, sleep duration, and sleep problems. *p < 0.05 for correlation, **p < 0.01 for correlation, ***p < 0.001 for correlation

The descriptive statistical analyses were performed using IBM SPSS (SPSS Inc., version 24.0). First, repeated measures ANOVAs were conducted to compare the differences in SDQ and sleep variables from T1 to T2 and from T2 to T3 and to study gender differences in sleep and emotional and behavioral difficulties. Second, Pearson correlations were calculated for examining the relations between sleep duration, sleep problems, and emotional and behavioral difficulties. To determine whether sleep duration and sleep problems predict adolescent emotional and behavioral difficulties or vice versa, cross-lagged structural equation models (SEM) were ran with Mplus (Mplus 8.3). In this study, data were used for participants who provided information either at T1 only (*n* = 8743; 51.1% girls), at both T1 and T2 (*n* = 5271; 51.6% girls), or at T1 and T3 (*n* = 3703; 54.0% girls), thus for around 27.4–64.8% of the age cohort. As adolescent socioeconomic status (SES) may play a role in physical health and sleep in adolescence (Marco et al. 2012), analyses were controlled for mothers’ educational level. Furthermore, because gender differences were expected to be found in adolescents’ sleep (Maslowsky and Ozer 2014), analyses were adjusted for gender. The structure of the SEM model was simplified by adding each component to the model as latent. This missing data method (missing at random approach) was used for estimating the model from full data without imputing. The cross-lagged path model for studying the relations between emotional and behavioral difficulties and sleep across the three measurement points is presented in Fig. 2. The model was fitted by applying maximum-likelihood estimation to use all available information instead of imputing data missing at random (Allison 2012). The model assessed the individual variation separately at T1, T2, and T3. All variables used in the model were normally distributed.

Goodness-of-fit was evaluated using the following descriptive indices: Comparative Fit Index (CFI), Tucker Lewis Index (TLI), and Root Mean Square Error of Approximation (RMSEA). Due to the large sample size, x^2^-test results are not reported here. First, full model was tested for sleep (sleep duration and sleep problems) and total difficulties on psychosocial behavior, including stability for sleep (T1–T3) and total difficulties (T1–T3), the paths from sleep at T1 to total difficulties at T2, the paths from sleep at T2 to total difficulties at T3, the paths from total difficulties at T1 to sleep at T2, and the paths from total difficulties at T2 to sleep at T3, among all participants. Finally, the full models for sleep and emotional symptoms, conduct problems, hyperactivity and peer problems were examined.

## Results

### Stability of Sleep and Emotional and Behavioral Difficulties during Adolescence

Repeated measures ANOVA revealed significant main effects for total difficulties, emotional symptoms, hyperactivity, conduct problems, and peer problems (Table 1). Adolescents’ sleep duration decreased over time, with 36 min less sleep from T1 to T2 and 13 min less sleep from T2 to T3 (*p* < 0.001). In addition, sleep problems increased significantly from T1 to T2 and from T2 to T3 (*p* < 0.001).

**Table 1:** Mean (M), standard deviation (SD), and repeated measures ANOVA *p*-values (*p*) for sleep, total difficulties on psychosocial behavior, emotional symptoms, conduct problems, hyperactivity, and peer problems at T1 (*n* = 8730–8743), T2 (*n* = 5271), and T3 (*n* = 3703)

	T1	T2	T3	ANOVA	
Variable (range)	M (SD)	M (SD)	M (SD)	*F*	*p*
Sleep duration, h:min	8:38 (0:46)	8:02 (0:48)	7:49 (0:46)	1228.31	<0.001
Sleep problems (0–4)	1.78 (1.02)	1.85 (1.02)	2.06 (1.02)	85.79	<0.001
Total difficulties on Psychosocial Behavior (0–40)	9.23 (5.32)	11.01 (5.88)	10.84 (5.65)	195.75	<0.001
Emotional symptoms (0–10)	2.44 (2.14)	3.11 (2.39)	3.32 (2.40)	204.35	<0.001
Conduct problems (0–10)	1.85 (1.58)	2.03 (1.76)	1.68 (1.60)	30.27	<0.001
Hyperactivity (0–10)	2.84 (2.12)	3.32 (2.28)	3.37 (2.28)	141.99	<0.001
Peer problems (0–10)	2.10 (1.66)	2.55 (1.79)	2.47 (1.69)	88.39	<0.001

Total difficulties increased significantly over time (*p* < 0.001). Further, emotional symptoms, hyperactivity, and peer problems increased throughout adolescence (*p* < 0.001 for all repeated measures ANOVAs). Conduct problems, on the other hand, increased from T1 to T2 (*p* < 0.001), but decreased from T2 to T3 (*p* < 0.001).

**Fig. 3:**
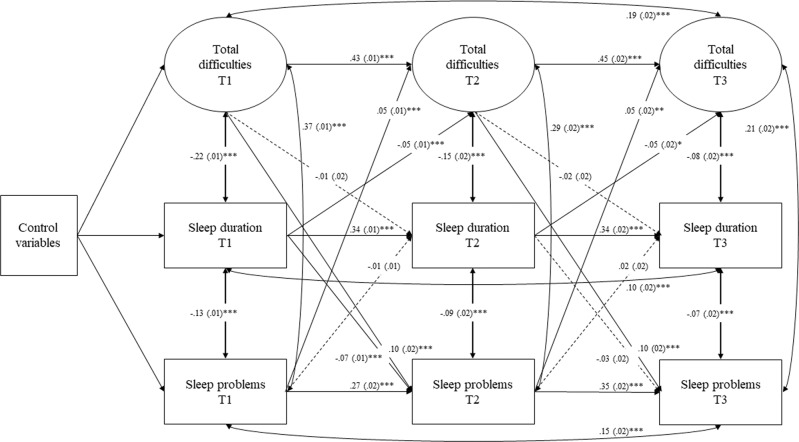
Mean scores for emotional and behavioral difficulties in adolescents who slept less than 8 h/night (*n* = 105, 59.0% girls) throughout adolescence as compared with adolescents who slept at least 8 h/night (*n* = 1036; 51.3% girls) throughout adolescence

Adolescents who slept less than 8 h throughout adolescence (*n* = 105, 59.0% girls) were also compared with adolescents who slept 8 h or more (*n* = 1036; 51.3% girls) at every measurement point. For both groups, emotional and behavioral difficulties measured by sum score for total difficulties on psychosocial behavior increased with age. However, adolescents who slept less than 8 h/night throughout adolescence had significantly more emotional and behavioral difficulties at T1 (*p* < 0.001), T2 (*p* < 0.001), and T3 (*p* < 0.001) than adolescents who slept at least 8 h/night at each measurement time (Fig. 3).

### Differences in Sleep and Emotional and Behavioral Difficulties by Gender

As shown in Table 2, girls had shorter sleep duration than boys at every time point. Girls also had more sleep problems than boys at each time point (*p* < 0.001). Regarding emotional and behavioral difficulties, the score for total difficulties was higher among girls than boys at each time point (*p* < 0.001). In addition, girls had more emotional problems than boys throughout adolescence (*p* < 0.001) and also more hyperactivity (*p* < 0.001). However, boys had more conduct problems (*p* < 0.001) and peer problems (*p* < 0.001) than girls throughout adolescence. Significant interaction effects were found for sleep duration, emotional symptoms, hyperactivity, and conduct problems. Therefore, the sleep duration of girls decreased more rapidly than that of boys (*p* < 0.001), the emotional symptoms and hyperactivity of girls increased more rapidly than that of boys (*p* < 0.001), and the conduct problems of boys changed more rapidly than that of girls (*p* < 0.001).

**Table 2:** Mean (M), standard deviation (SD), and repeated measures ANOVA *p*-values (*p*) for sleep duration, sleep problems, total difficulties on psychosocial behavior, emotional symptoms, conduct problems, hyperactivity, and peer problems in boys and girls at T1 (*n* = 8730–8743; 51.1% girls) or at T1 and T2 (*n* = 5271; 51.6% girls) or at T1 and T3 (*n* = 3703; 54.0% girls)

	T1 M (SD)		T2 M (SD)		T3 M (SD)			ANOVA	
Variable (range)	Boys	Girls	Boys	Girls	Boys	Girls		*F*	*p*
Sleep duration, h:min	8:44 (0:47)	8:32 (0:45)	8:05 (0:50)	7:58 (0:46)	7:49 (0:45)	7:49 (0:46)		12.72	<0.001
							Interaction:	12.22	<0.001
Sleep problems (0–4)	1.64 (0.96)	1.91 (1.06)	1.68 (0.95)	2.01 (1.05)	1.88 (0.97)	2.21 (1.04)		111.31	<0.001
							Interaction:	2.98	0.051
Total difficulties on psychosocial behavior (0–40)	8.76 (5.32)	9.68 (5.29)	10.56 (6.17)	11.44 (5.56)	10.19 (5.83)	11.39 (5.43)		34.26	<0.001
							Interaction:	1.66	0.191
Emotional symptoms (0–10)	1.79 (1.87)	3.06 (2.21)	2.33 (2.16)	3.85 (2.37)	2.55 (2.16)	3.98 (2.39)		414.90	<0.001
							Interaction:	2.91	<0.001
Conduct problems (0–10)	1.97 (1.66)	1.74 (1.50)	2.30 (1.91)	1.78 (1.56)	1.82 (1.76)	1.56 (1.45)		42.78	<0.001
							Interaction:	10.89	<0.001
Hyperactivity (0–10)	2.75 (2.07)	2.93 (2.17)	3.20 (2.18)	3.43 (2.36)	3.13 (2.16)	3.56 (2.35)		14.43	<0.001
							Interaction:	6.00	<0.01
Peer problems (0–10)	2.25 (1.73)	1.96 (1.58)	2.73 (1.88)	2.37 (1.69)	2.68 (1.74)	2.29 (1.63)		44.16	<0.001
							Interaction:	1.72	0.179

### Cross-Sectional Correlations between Sleep and Emotional and Behavioral Difficulties across Adolescence

Table 3 presents Pearson correlations between sleep duration, general sleep problems, total difficulties, emotional symptoms, conduct problems, hyperactivity and peer problems at T1, T2, and T3. Bivariate correlations (T1–T3) showed that shorter sleep duration correlated with more total difficulties (*r* = −0.04 to −0.20, *p<* 0.01 for all correlations), emotional symptoms (*r* = −0.04 to −0.14, *p<* 0.01 for all correlations), conduct problems (*r* = −0.04 to −0.22, *p<* 0.01 for all correlations), and hyperactivity (*r* = −0.06 to −0.22, *p<* 0.01 for all correlations) throughout adolescence, both at the measurement age and contributing to later emotional and behavioral difficulties. Regarding peer problems, shorter sleep duration at either T2 or T3 correlated significantly with more peer problems at T3 (sleep duration at T2: *r* = −0.06, *p<* 0.01 and at T3: *r* = −0.05, *p<* 0.05). Vice versa, T2 peer problems correlated with shorter sleep duration at T3 (*r* = −0.04, *p<* 0.05).

**Table 3:** Pearson correlations between sleep duration, sleep problems, and emotional and behavioral difficulties at T1, T2, and T3

Variable	1	2	3	4	5	6	7	8	9	10	11	12	13	14	15	16	17	18	19	20
1. T1: Sleep duration																				
2. T2: Sleep duration	0.35^b^																			
3. T3: Sleep duration	0.25^b^	0.37^b^																		
4. T1: Sleep problems	−0.12^b^	−0.06^b^	−0.07^b^																	
5. T2: Sleep problems	−0.09^b^	−0.09^b^	−0.02	0.34^b^																
6. T3: Sleep problems	−0.07^b^	−0.08^b^	−0.08^b^	0.29^b^	0.43^b^															
7. T1: Total difficulties on Psychosocial Behavior	−0.20^b^	−0.08^b^	−0.10^b^	0.36^b^	0.22^b^	0.16^b^														
8. T2: Total difficulties on Psychosocial Behavior	−0.12^b^	−0.17^b^	−0.08^b^	0.20^b^	0.39^b^	0.26^b^	0.47^b^													
9. T3: Total difficulties on Psychosocial Behavior	−0.11^b^	−0.14^b^	−0.12^b^	0.18^b^	0.27^b^	0.32^b^	0.42^b^	0.55^b^												
10. T1: Emotional symptoms	−0.12^b^	−0.04^a^	−0.08^b^	0.37^b^	0.24^b^	0.19^b^	0.75^b^	0.35^b^	0.33^b^											
11. T2: Emotional symptoms	−0.12^b^	−0.14^b^	−0.07^b^	0.21^b^	0.41^b^	0.30^b^	0.38^b^	0.77^b^	0.46^b^	0.47^b^										
12. T3: Emotional symptoms	−0.11^b^	−0.10^b^	−0.08^b^	0.18^b^	0.27^b^	0.36^b^	0.32^b^	0.42^b^	0.76^b^	0.41^b^	0.57^b^									
13. T1: Conduct problems	−0.22^b^	−0.08^b^	−0.09^b^	0.22^b^	0.12^b^	0.06^b^	0.71^b^	0.33^b^	0.26^b^	0.32^b^	0.14^b^	0.10^b^								
14. T2: Conduct problems	−0.09^b^	−0.13^b^	−0.04^b^	0.10^b^	0.20^b^	0.09^b^	0.29^b^	0.71^b^	0.33^b^	0.10^b^	0.33^b^	0.09^b^	0.38^b^							
15. T3: Conduct problems	−0.07^b^	−0.10^b^	−0.11^b^	0.06^b^	0.11^b^	0.12^b^	0.25^b^	0.34^b^	0.68^b^	0.09^b^	0.15^b^	0.30^b^	0.31^b^	0.41^b^						
16. T1: Hyperactivity	−0.22^b^	−0.09^b^	−0.07^b^	0.22^b^	0.16^b^	0.11^b^	0.73^b^	0.40^b^	0.33^b^	0.33^b^	0.21^b^	0.15^b^	0.53^b^	0.31^b^	0.26^b^					
17. T2: Hyperactivity	−0.13^b^	−0.17^b^	−0.06^b^	0.15^b^	0.26^b^	0.16^b^	0.36^b^	0.72^b^	0.43^b^	0.18^b^	0.35^b^	0.21^b^	0.29^b^	0.47^b^	0.29^b^	0.48^b^				
18. T3: Hyperactivity	−0.08^b^	−0.12^b^	−0.11^b^	0.14^b^	0.20^b^	0.21^b^	0.32^b^	0.43^b^	0.72^b^	0.18^b^	0.25^b^	0.33^b^	0.22^b^	0.29^b^	0.43^b^	0.40^b^	0.56^b^			
19. T1: Peer problems	0.01	−0.01	−0.05^a^	0.16^b^	0.08^b^	0.07^b^	0.58^b^	0.23^b^	0.22^b^	0.35^b^	0.18^b^	0.18^b^	0.21^b^	0.05^a^	0.05^b^	0.12^b^	0.01	0.05^a^		
20. T2: Peer problems	0.01	−0.03	−0.04^a^	0.10^b^	0.17^b^	0.14^b^	0.30^b^	0.61^b^	0.32^b^	0.20^b^	0.37^b^	0.23^b^	0.13^b^	0.31^b^	0.13^b^	0.10^b^	0.14^b^	0.09^b^	0.42^b^	
21. T3: Peer problems	−0.03	−0.06^b^	−0.04	0.08^b^	0.12^b^	0.14^b^	0.26^b^	0.33^b^	0.60^b^	0.15^b^	0.22^b^	0.36^b^	0.13^b^	0.18^b^	0.32^b^	0.11^b^	0.10^b^	0.13^b^	0.35^b^	0.50^b^

^a^Correlation is significant at 0.05 level

^b^Correlation is significant at 0.01 level

Self-reported sleep problems were also related to emotional and behavioral difficulties throughout adolescence, similarly to shorter sleep duration. The more self-reported sleep problems present at T1, T2, and T3, the more total difficulties (*r=* 0.32 to 0.39, *p* < 0.01 for all correlations), emotional symptoms (*r* = 0.36 to 0.41, *p* < 0.01 for all correlations), conduct problems (*r=* 0.12 to 0.22, *p* < 0.01 for all correlations), hyperactivity (*r=* 0.21 to 0.26, *p* < 0.01 for all correlations), and peer problems (*r=* 0.14 to 0.17, *p* < 0.01 for all correlations) arose throughout adolescence, both at the measurement age and contributing to later emotional and behavioral difficulties.

### Cross-Lagged Models for Sleep and Emotional and Behavioral Difficulties

The full model for total difficulties and sleep fitted the data well (RMSEA = 0.02, SRMR = 0.01, CFI = 1.0, TLI = 0.99). Figure 2 shows the cross-sectional and longitudinal pathways in SEM model for total difficulties and sleep variables. From a longitudinal perspective, short sleep duration (at T1 or T2) was associated with slightly more total difficulties later in adolescence (at T2: β = −0.05, *p* < 0.001 [95% CI, −0.07 to −0.02]; at T3: β = −0.05, *p* < 0.05 [95% CI, −0.09 to −0.01]). Further, sleep problems (at T1 or T2) were associated with slightly more total difficulties later in adolescence (at T2: β = 0.05, *p* < 0.001 [95% CI, 0.03–0.08]; at T3: β = 0.05, *p* < 0.01 [95% CI, 0.02–0.09]). Vice versa, total difficulties (at T1 or T2) were associated with slightly more sleep problems later in adolescence (at T2: β = 0.10, *p* < 0.001 [95% CI, 0.08–0.13]; at T3: β = 0.10, *p* < 0.001 [95% CI, 0.06–0.13]). However, total difficulties were not associated with sleep duration later in adolescence.

### Cross-Lagged Models for Sleep, Emotional Symptoms, Conduct Problems, Hyperactivity and Peer Problems

The full models for emotional symptoms, conduct problems, hyperactivity, peer problems and sleep duration and sleep problems fit the data well. Table 4 shows the fit indices for each model. First, the model for sleep and emotional symptoms was examined. The full model for the emotional symptoms and sleep variables fit the data well (RMSEA = 0.02, SRMR = 0.01, CFI = 0.99, TLI = 0.97). Short sleep duration at T1 was associated with slightly more emotional symptoms at T2 (β = −0.04, *p* < 0.05 [95% CI, −0.07 to −0.02]), but the association was not significant from T2 to T3 (β = −0.03, *p* > 0.05 [95% CI, −0.06–0.01]). Sleep problems either at T1 or T2 were associated with more emotional symptoms later in adolescence (at T2: β = 0.05, *p* < 0.001 [95% CI, 0.02–0.08]; at T3: β = 0.04, *p* < 0.01 [95% CI, 0.00–0.08]). Vice versa, emotional symptoms either at T1 or T2 were associated with more sleep problems later in adolescence (at T2: β = 0.15, *p* < 0.001 [95% CI, 0.12–0.18]; at T3: β = 0.14, *p* < 0.001 [95% CI, 0.10–0.17]). However, emotional symptoms were not associated with sleep duration later during adolescence.

**Table 4:** Model fit statistics

Model	RMSEA	SRMR	CFI	TLI
Criteria for acceptable fit	<0.06	<0.07	>0.95	>0.90
Sleep and total difficulties on psychosocial behavior	0.015	0.007	0.998	0.985
Sleep and emotional symptoms	0.024	0.014	0.993	0.965
Sleep and conduct problems	0.025	0.016	0.988	0.943
Sleep and hyperactivity	0.024	0.014	0.991	0.956
Sleep and peer problems	0.027	0.017	0.986	0.935

Second, the model for sleep and conduct problems was examined. The full model for the conduct problems and sleep duration and sleep problems fit the data well (RMSEA = 0.03, SRMR = 0.02, CFI = 0.99, TLI = 0.94). Short sleep duration at T2 was associated with more conduct problems at T3 (β = −0.05, *p* < 0.001 [95% CI, −0.08 to −0.01]). Conversely, conduct problems were not associated with later sleep duration. Conduct problems at T1, however, were associated with more sleep problems at T2 (β = 0.05, *p* < 0.001 [95% CI, 0.02–0.08]). Sleep problems were not associated with conduct problems later in adolescence.

Third, the model for sleep and hyperactivity was examined. The full model for the hyperactivity and sleep fit the data well (RMSEA = 0.02, SRMR = 0.01, CFI = 0.99, TLI = 0.96). Short sleep duration at T1 was associated with more hyperactivity at T2 (β = −0.03, *p* < 0.05 [95% CI, −0.05–0.00]). However, hyperactivity was not associated with sleep duration later during adolescence. Further, sleep problems either at T1 or T2 were associated with more hyperactivity later in adolescence (at T2: β = 0.06, *p* < 0.001 [95% CI, 0.04–0.09]; at T3: β = 0.06, *p* < 0.01 [95% CI, 0.02–0.09]). Hyperactivity either at T1 or T2 was associated with more sleep problems later in adolescence (at T2: β = 0.08, *p* < 0.001 [95% CI, 0.06–0.11]; at T3: β = 0.06, *p* < 0.01 [95% CI, 0.02–0.09]).

Last, the model for sleep and peer problems was examined. The full model for the peer problems and sleep variables fit the data well (RMSEA = 0.03, SRMR = 0.02, CFI = 0.99, TLI = 0.94). Sleep problems either at T1 or T2 were associated with more peer problems later in adolescence (at T2: β = 0.03, *p* < 0.05 [95% CI, 0.01–0.06]; at T3: β = 0.04, *p* < 0.05 [95% CI, 0.00–0.07]). Vice versa, peer problems at T2 were associated with more sleep problems (β = 0.05, *p* < 0.01 [95% CI, 0.02–0.09]) and shorter sleep duration (β = −0.04, *p* < 0.05 [95% CI, −0.08 to −0.01]) at T3, but there were no significant longitudinal associations between peer problems and sleep variables from T1 to T2. Autoregressive models have been strongly criticized (Berry and Willoughby 2017). An alternative model proposed in the article of Hamaker et al. (2015) was tested. If constructs in the model are trait-like, the lagged parameters do not represent the actual within-person relationships over time, which can lead to mistaken conclusions (Hamaker et al. 2015). To see if the results would change the within-person process was separated from stable between-person differences through inclusion of random intercepts. However, the results stay the same unless we add an association between the random intercepts for sleep and internalizing/externalizing behavior, respectively.

## Discussion

The role of sleep in mental health during adolescence is well established in cross-sectional studies. However, the longitudinal and bidirectional relationship between sleep and psychosocial well-being are largely unknown (Gregory and Sadeh 2012). Hence, longitudinal approach is required in order to understand the complex interplay between sleep and mental health and their long-term effects on well-being. Moreover, few studies have been conducted on the associations between sleep and emotional and behavioral difficulties in late adolescence (Becker et al. 2015; Merikanto et al. 2017) which is a developmental period sensitive to sleep disturbances (Crowley et al. 2007) and mental health problems (Keyes 2006) and their reciprocal associations (Pieters et al. 2015).

This study aimed to investigate the relation between self-reported sleep duration, sleep problems, and emotional and behavioral difficulties during adolescence. The longitudinal bidirectional links between sleep and emotional and behavioral difficulties were evaluated in three different age groups throughout adolescent years, a topic noted in previous research to have gaps in knowledge (Gregory and Sadeh 2012). In the present study, the bidirectionality of these associations was examined across five years in adolescence in a large longitudinal sample to determine whether sleep is associated with current as well as prospective emotional and behavioral difficulties, and vice versa. The findings of the present study showed that short sleep duration was a stronger predictor of current and later increased emotional and behavioral difficulties than vice versa throughout adolescence. On the other hand, increased emotional and behavioral difficulties predicted current and future sleep problems more strongly than reverse. Overall, short sleep duration predisposed to emotional and behavioral difficulties throughout adolescence followed by prospective sleep problems.

In this study, the influence of sleep duration and sleep problems on current emotional and behavioral difficulties was reflected in the total difficulties on psychosocial behavior and in emotional symptoms, conduct problems, hyperactivity and peer problems at each of the three time point from 12 to 17 years of age. Relative to adolescents with longer sleep duration and less sleep problems, adolescents with shorter sleep duration and more sleep problems had increased total difficulties on psychosocial behavior, emotional symptoms, hyperactivity, peer problems, and conduct problems. These results are in line with previous findings indicating that insufficient sleep is associated with somewhat weakened adolescent daytime functioning (Liu and Zhou 2002). Furthermore, the finding that short sleep duration is associated with externalizing problem behavior, such as hyperactivity, peer problems, and conduct problems, is in accord with an earlier study on adolescents that suggested that short sleep duration could play a role in the association between eveningness and externalizing problem behavior (Merikanto et al. 2017). Consistent with previous research findings (Asarnow et al. 2014), short sleep duration predicted emotional difficulties, which predicted subsequent sleep problems. In line with the present results on the bidirectional relation between sleep and emotional and behavioral problems in adolescents, depression studies have shown bidirectional associations between insufficient sleep and depression in adolescence. Short sleep can increase depressive symptoms, which then increase the risk for sleep problems (Alvaro et al. 2013). In addition, the findings of this study suggest that adolescents who sleep less than 8 h/night throughout adolescence have more emotional and behavioral difficulties at each measurement time than their peers who sleep by average systematically at least 8 h/night. Emotional and behavioral difficulties earlier in adolescence were not, however, associated with shorter sleep duration later in adolescence. This finding was also reported by Quach et al. (2018) who found sleep to be a significant predictor for later internalizing difficulties, but internalizing problems not to be a significant predictor for later sleep. In line with the results of Quach et al. (2018), this study confirms that sleep is an important factor for emotional symptoms and behavioral outcomes during different school transitions.

The associations between self-reported sleep problems and emotional and behavioral difficulties were reciprocal, showing that sleep problems in early or middle adolescence were associated with slightly increased emotional and behavioral difficulties, emotional symptoms, conduct problems, hyperactivity, and peer problems in middle or late adolescence and vice versa. Peer problems in early or middle adolescence, on the other hand, predicted both sleep problems and short sleep duration later in adolescence. Previous studies have suggested that sleep problems during childhood/adolescence can predispose to internalizing difficulties later in life (Kechter and Leventhal 2018, and childhood psychological factors, such as emotional security and poor child-parent relationships, can lead to sleep problems (Keller and El-Sheikh 2011). Consistent with the literature, this research supports earlier findings that the associations between sleep and emotional well-being are bidirectional rather than one process explaining the development of the other (Pieters et al. 2015). In conclusion, according to the cross-lagged models for sleep and emotional and behavioral difficulties, these findings indicate a developmental process where short sleep duration is a stronger predictor for emotional and behavioral difficulties than vice versa. Emotional and behavioral difficulties, on the other hand, predict later sleep problems more strongly than the converse.

Yet, no previous research has tackled in depth the development of both internalizing and externalizing difficulties from early to late adolescence. With a five-year follow-up of adolescents from the mean age of 13 to 17 years of age, the novel findings of the present study demonstrate that emotional symptoms (e.g. psychosomatic symptoms, worrying, feelings of sadness and worthlessness) and hyperactivity increased throughout adolescence, whereas total difficulties on psychosocial behavior increased from early to middle adolescence, but not from middle adolescence to late adolescence. Peer problems only increased from early to middle adolescence. Conduct problems were infrequent in this study and increased from early to middle adolescence, followed by a decrease from middle to late adolescence. During early adolescence, the importance of peers grows and the dependency on parents decreases. Therefore, friendships begin to have more effect on the behavior of the individual (Wilkinson 2008). The decrease of conduct and peer problems could be explained by the change in social behavior, e.g. the growing meaning of friends and peers, from middle to late adolescence. As there are no previous research studying the development of these problems from early to late adolescence in similar depth as the present study, we cannot compare the results presented here on the changes in psychosocial symptoms across early to late adolescence to previous research.

The results of this study are in line with a previous study on Finnish adolescents (Kuula et al. 2018), as throughout adolescence findings here showed that the amount of sleep problems increased while sleep duration decreased. In line with previous studies (Brand et al. 2014), the findings of this study show that sleep duration is shorter in late adolescence than in early and middle adolescence, decreasing gradually from early to late adolescence. The National Sleep Foundation recommends 8 to 10 h of sleep each night for adolescents to function best (Hirshkowitz et al. 2015). Since sleep is particularly important not only for various factors of health and well-being but also for adolescent brain maturation (Dahl and Lewin 2002) and psychosocial well-being (Kalak et al. 2014), it is important to follow The National Sleep Foundation sleep guidelines. Average sleep durations in the present study at different measurement ages were similar to those reported previously in a national United States sample, where adolescents adolescence slept the average of 8.5 h per night at age 13 and the average of 7.3 h at age 18 (Maslowsky and Ozer 2014). Average adolescent sleep durations in the present study were however lower than in a normative Australian sample, where adolescents at ages 16–17 had an average sleep duration of approximately 9 h (Olds et al. 2010). Around 10% of the adolescents slept less than 8 h throughout adolescence in the present study presenting a risk group for poorer psychosocial well-being and impaired development.

In this study, girls had shorter sleep duration than boys in early and middle adolescence, which supports previous research findings (e.g. Patte et al. 2018). According to previous studies, as compared to boys, girls sleep more than males in late adolescence and early adulthood (Maslowsky and Ozer 2014) and report more sleep problems (Kechter and Leventhal 2018) during adolescence. Here there were no differences in sleep duration between girls and boys in late adolescence. Since the sleep duration of boys decreased more from middle to late adolescence as compared to girls, the finding can demonstrate the developmental trajectory of decreasing sleep duration in adolescent boys on the threshold of emerging adulthood. Supporting previous findings (Kechter and Leventhal 2018), girls also had more sleep problems than boys across adolescence. Further, the findings of the present study show that girls had more total difficulties on psychosocial behavior than boys throughout adolescence. In addition, girls had more emotional symptoms and reported more hyperactivity than boys throughout adolescence. However, boys had more conduct problems and peer problems than girls. Previous research has shown differences in girls and boys concerning the quality and appearance of mental health problems (Wang et al. 2015). A recent study reported Finnish adolescent girls as having less conduct problems than boys, but, in contrast to the findings of this study, also less hyperactivity than boys (Mishina et al. 2018). Being consistent with previous findings (Fink et al. 2015; Van Roy et al. 2006), girls had more emotional problems and less conduct and peer problems (also occurring later in adolescence) than boys. However, in contrast to what is found earlier (Fink et al. 2015), girls were more hyperactive than boys in this study. The underlying reasons for the differences in emotional and behavioral difficulties between girls and boys may be explained by the finding that girls sleep less than boys and have more sleep problems across adolescence. Other studies have reported an increase in internalizing problems in adolescent girls over the last 10 years (Bor et al. 2014). Gender differences in emotional and behavioral difficulties that emerged in this study might be explained by the fact that girls confront more sleep problems and sleep less as compared to boys during adolescence. Another potential explanation could be that different genders face different demands in society. Compared with boys, adolescent girls seem to have more demands imposed on them by society, especially in school (Salmela-Aro and Tynkkynen 2012).

The main strength of this study is the large-scale longitudinal data from one age cohort in the Helsinki metropolitan area. However, some limitations must be noted. In addition, all data were gathered by self-reported questionnaires; thus, the assessment of emotional and behavioral difficulties and sleep was subjective. However, in this study bedtime and wake-up time are self-reported averages over time, whereas objective sleep measures are momentary estimations for sleep. When it comes to examining age groups at the population level comprehensive sampling with objective measures is challenging to conduct. Furthermore, an objective measurement would not have yielded such a large sample size, reflecting well the Finnish children and adolescents at a population-based level. Sleep duration was measured by counting the hours between bedtime and wake-up time. The anchors of bedtime and wake-up time were used to determine only estimated sleep duration. Since the first and last response options for bedtime and wake-up time were open-ended, the variable measuring self-reported sleep duration is merely indicative. In addition, Cronbach’s alphas for conduct and peer problems were low at every time point. As the research was conducted as part of the school routine, information was collected only on students on the target grade who were present during the school day. Therefore, the results could have been different had the follow-up sample included everyone who participated in T1.

## Conclusion

The complex associations between sleep and psychosocial development from early to late adolescence are still largely unknown. A longitudinal approach is needed to understand the interplay between sleep and psychosocial outcomes and their long-term effects on well-being. With a five-year follow-up of Finnish adolescents, this longitudinal study examined the relations between self-reported sleep duration, sleep problems, and psychosocial well-being during adolescence. This study showed a reciprocal path between disturbed sleep and emotional and behavioral difficulties. On one hand, short sleep duration across adolescence predisposes to various psychosocial difficulties including emotional symptoms, conduct problems and hyperactivity. On the other hand, emotional and psychosocial difficulties across adolescence can predispose to sleep problems later in adolescence. The aforementioned novel findings of the present study shed light on the associations between sleep and emotional and behavioral difficulties drawing a more complex picture than one is the cause of the other. Furthermore, this longitudinal study suggests that the interplay between sleep and emotional and behavioral difficulties not only affects the current state of well-being but also predicts future sleep and emotional and behavioral difficulties during adolescence. Together, these findings indicate that sleep plays a significant role in adolescent externalizing and internalizing behaviors, and vice versa as emotional and behavioral difficulties influence adolescent sleep quality. The results of this study indicate that adolescent sleep should be considered more extensively in order to give advice for planning adolescent health promotion programs.
